# Perceptions of Individual and Community Environmental Influences on Fruit and Vegetable Intake, North Carolina, 2004

**Published:** 2008-12-15

**Authors:** Josephine E. A. Boyington, Britta Schoster, Jack Shreffler, Kathryn Remmes Martin, Leigh F. Callahan

**Affiliations:** NIH/NINR. Dr Boyington's contribution to this article occurred in association with her tenure as a research associate/epidemiologist at Shaw University and as a Diversity Research investigator at Thurston Arthritis Research Center, University of North Carolina at Chapel Hill; Thurston Arthritis Research Center; Thurston Arthritis Research Center; Thurston Arthritis Research Center, Gillings School of Public Health, the Institute on Aging; Thurston Arthritis Research Center, Department of Medicine, Department of Orthopedics, Department of Social Medicine, University of North Carolina at Chapel Hill

## Abstract

**Introduction:**

Increases in obesity and other chronic conditions continue to fuel efforts for lifestyle behavior changes. However, many strategies do not address the impact of environment on lifestyle behaviors, particularly healthy dietary intake. This study explored the perceptions of environment on intake of fruits and vegetables in a cohort of 2,479 people recruited from 22 family practices in North Carolina.

**Methods:**

Participants were administered a health and social demographic survey. Formative assessment was conducted on a subsample of 32 people by using focus groups, semistructured individual interviews, community mapping, and photographs. Interviews and discussions were transcribed and content was analyzed using ATLAS.ti version 5. Survey data were evaluated for means, frequencies, and group differences.

**Results:**

The 2,479 participants had a mean age of 52.8 years, mean body mass index (BMI) of 29.4, and were predominantly female, white, married, and high school graduates. The 32 subsample participants were older, heavier, and less educated. Some prevalent perceptions about contextual factors related to dietary intake included taste-bud fatigue (boredom with commonly eaten foods), life stresses, lack of forethought in meal planning, current health status, economic status, the ability to garden, lifetime dietary exposure, concerns about food safety, contradictory nutrition messages from the media, and variable work schedules.

**Conclusion:**

Perceptions about intake of fruits and vegetables intake are influenced by individual (intrinsic) and community (extrinsic) environmental factors. We suggest approaches for influencing behavior and changing perceptions using available resources.

## Introduction

Lifestyle practices of unhealthy diet and physical inactivity are noted determinants of chronic conditions, especially overweight and obesity (1). Nationally, chronic disease statistics document that the southeastern US region manifests some of the highest rates of and the worst outcomes for chronic disease (2). North Carolina is representative of the southeastern region; its age-adjusted death rates for the 5 diseases that account for almost two-thirds of all annual deaths are higher than the national average (3). Furthermore, the combined prevalence of adult overweight and obesity is 63% (3). This pattern of statistics is evidence of poor access to health services, poor economic opportunities, unique environmental challenges, and unhealthy lifestyle practices prevalent in this US region (3-6).

### Context

In North Carolina, approximately 77% of adults do not consume the recommended daily intake of 5 or more servings of fruits and vegetables and 26% do not engage in leisure time physical activity (3,4). These statistics underscore the prevalence of unhealthy behaviors and chronic conditions in this US region, yet provide little information about the motivations and perceptions of residents relative to these factors. Especially important is the paucity of data on peoples' qualitative descriptions of their dietary behaviors in the context of their environment. This is of particular interest given that lifestyle choices, including those relating to diet, are complex decisions affected by the interaction between people (attitudes, thoughts, behaviors, perceptions) and their external social and physical environments (7-9).

### Conceptual/theoretical framework

Historically, most assessments of dietary and other lifestyle behaviors and attitudes have been conducted using survey methods. However, because of their systematic structure, surveys have been less able to contextualize individuals' experiences and perceptions and do not facilitate easy assessment of motivational factors and contexts underpinning peoples' behaviors (10). Nonetheless, they have been useful for identifying patterns of behavior and facilitating comparisons of patterns across environments (10). However, interventions based on survey results, primarily individual-focused education strategies, have been limited in effecting behavior change and improving health outcomes (11). Researchers suggest that the absence of corresponding environmental changes may be responsible for this outcome (11). In fact, despite numerous preventive efforts regarding dietary behavior and chronic diseases, obesity and other chronic conditions continue to increase in prevalence. This change indicates that the mere identification of unhealthy behaviors and development of educational strategies to change them may not be enough. Researchers often state that people's behaviors are contextual and must therefore be understood and addressed within their environment (7,8,9,11-13). Yet, few studies have investigated individuals' perceptions of the relationship between their environment and diet and physical activity behaviors (11). Until recently, no unifying conceptual framework existed to evaluate these relationships. However, a work group of researchers has produced such a framework. The work group summarizes its effort as the beginning phase in clarifying the relationship between the environment and lifestyle behaviors and strongly encourages further inquiries on "the extent of environmental influence and how [environment and lifestyle] affect different individuals" (11).

An approach that has proved useful in both identifying and contextualizing lifestyle behaviors is formative research. This unique multimethod, exploratory approach combines social science techniques such as interviews, focus groups, and other qualitative methods with quantitative methods such as surveys (14). When these techniques are used to gather data for informing research conduct and intervention design, the term *formative assessment* is used. Formative research has been used primarily by intervention researchers in community-based studies and has guided the development of several interventions (13,15,16). A unique strength of this approach is that it provides the basis for collection of relevant new data to guide research direction, research discourse, and data interpretation (13,15-17). Drawing on a large study to assess community and social determinants of chronic disease risk and outcomes among North Carolina adults, we used formative research to report specifically on findings relative to fruit and vegetable intake.

## Methods

### Study design and sample description

The study proceeded in 2 phases: a telephone survey followed by a formative assessment phase. For the formative assessment, the research protocol included focus groups, semistructured individual interviews, photographs, and cursory community mappings.

Study participants were residents of North Carolina who had previously consented to be part of a unique statewide practice-based research network called the North Carolina Family Medicine Research Network (NC-FM-RN) (18). The NC-FM-RN was initiated in 2001 and currently includes a cohort of North Carolina residents previously recruited through a network of 25 family practice settings across the state. The cohort was enriched with new participants in 2004 and 2005. The sample for this study included people who were part of the cohort in 2001 and 2004. They were recruited from 22 of the 25 sites. Further details on the structure, recruitment methods, and outcomes of the NC-FM-RN are available elsewhere (18). All the methods and the protocols for this study and the NC-FM-RN were approved by the University of North Carolina at Chapel Hill Medical Institutional Review Board.

### Survey procedures and measures

At the study's outset in 2004-2005, previously enrolled participants from 22 of the 25 practices in the NC-FM-RN were sent a letter that described the study, its objectives, what participants could expect, and reminded participants that they had previously consented to be contacted for future studies. Consenting participants were scheduled for the telephone survey, which included a 30-minute assessment of demographic, health, and social factors, and health attitudes, behaviors, and beliefs related to chronic diseases. A total of 2,479 people from the cohort participated in the telephone survey, resulting in a 59.5% response rate. All measures in the survey were self-reported. Race was identified as non-Hispanic black, non-Hispanic white, or other (inclusive of all other races). Comorbidity was measured as reported diagnosis of 1 or more of 18 chronic conditions. Some of the conditions assessed were heart disease, hypertension, diabetes, and depression. Comorbidity scores were calculated by summing the number of reported diagnoses. Body mass index (BMI) was calculated from self-reported weight and height, and was reported as weight in kilograms/(height in meters)^2^. It was categorized according to Centers for Disease Control and Prevention guidelines (underweight, <18.5; normal weight, 18.5-24.9; overweight, 25.0-29.9; and obese, ≥30.0) (19). The survey also included the following 3 questions to assess participants' perception of the quality, variety, and affordability of fruits and vegetables in their environment:

- Thinking of the store where you do most of your grocery shopping, how would you rate the quality of their fresh fruits and vegetables?
- How would you rate the variety of their fresh fruits and vegetables?
- How would you rate the affordability of their fresh fruits and vegetables?

The response options for the first 2 questions ranged from *excellent *to *not applicable *and for the third question,* very affordable *to* not applicable.*

### Formative assessment

Participants were eligible to participate in focus group discussions if they had completed the telephone survey. Six of the 22 NC-FM-RN practices were chosen as recruitment sites, and patients from these sites were invited to participate first by letter and then by telephone calls. A total of 84 people were recruited, and 21 (25%) attended the discussion sessions. Seven focus groups, averaging 3 participants and lasting 1.5 hours, were conducted in 6 locations. Participants received a gift of $20. Sessions were arranged, facilitated, and audiotaped with participants' consent by 2 trained moderators (B.S., L.F.C.) and subsequently transcribed, coded, and analyzed (B.S., K.R.M.) using ATLAS.ti version 5.0 software (Scientific Software Development, GmbH, Berlin, Germany). Nutrition-specific thematic content and coding analyses were further conducted (J.E.A.B.). Portions of the transcripts that addressed participants' reported consumption patterns of fresh fruits and vegetables and their perceptions of environmental influences on their nutrition behaviors were reviewed for emergent themes and compared across groups. Participants responded to the question, "Do you think about getting fruit and vegetables in your diet?"

### Semistructured individual interviews

To broaden the range of perspectives, semistructured individual interviews were conducted with community members not demographically represented in the focus groups. These individuals were recruited from the same list used for the focus group participants. A total of 11 participants who were African American men, African American women, or white men were intentionally recruited to participate in telephone-administered interviews. This addition increased the formative assessment sample to 32.

### Community mapping

Before each focus group session, a cursory geographic assessment of each of the 6 localities was conducted. Research team members drove through the downtown sections of localities on the day of the session and identified physical assets that could serve as resources for or constraints to lifestyle behavior change. Relevant assets included the availability, type, and proximity of grocery stores, farm stands, restaurants, and convenience stores. This endeavor helped moderators to contextualize the behaviors and perceptions reported by the participants during the sessions.

### Photography

Participants were asked to identify and photograph factors in their environment that they perceived demonstrated a relationship between their community and health. They were each given a disposable camera and a photo-diary with instructions on how to document their thoughts about their photographs. They were also instructed that the purpose of this activity was to facilitate discussion and that the activity was not a requirement for participation in the discussions. Submitted photographs were coded as representative of barriers or facilitators if participants showed them to the group in their responses to nutrition questions or if the photographs noticeably related to nutrition.

### Plan of analysis

To identify demographic characteristics and group differences, survey results were analyzed using univariate and bivariate statistics. Differences in perceptions of affordability, accessibility, and variety of fruits and vegetables in neighborhood stores were tested by categories of race, BMI, socioeconomic status (SES) as determined by income and poverty rate by census block group, education, and chronic disease status using χ^2^ test of differences at the α = .05 level. The poverty rate by census block group variable was created by using 2000 census data, which indicated that 12.3% of the North Carolina population lives below the poverty level. Focus group transcripts were content-analyzed for prevalent perceptions related to fruit and vegetable intake (20,21). Identified perceptions were then categorized by using the conceptual framework proposed by Booth and colleagues (11) (Figure, Table 1).

**Figure 1:**
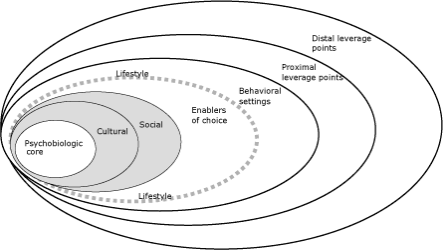
Framework of determinants of physical activity and eating behavior, from Booth et al (11). Reprinted with permission.

The framework includes individual (intrinsic), lifestyle-enabling, and external (extrinsic) environmental factors. Pictorially (Figure), the framework consists of 7 concentric rings categorized from the innermost to the outermost ring as follows: *psychobiologic core, cultural, social, enablers of choice, behavioral settings, proximal leverage points,* and *distal leverage points.* Each ring consists of specific factors that are separate from those in other rings (further described in Table 1). The individual's environment includes the psychobiologic core and cultural and social segments. The *psychobiologic core* consists of the individual's current physical health status and encompasses factors such as genes, instinctive and conditioned behaviors, and experiential learning. The *cultural *segment encompasses values, traits, and beliefs acquired from the social and cultural environment. The *social *segment covers how people perceive themselves and their social roles, and includes acquired beliefs and values (11). Positioned between the individual and the external environments are the *enablers of choice*. These are the barriers and facilitators to change that are proximal to the individual and include factors that facilitate interaction between the individual and the external environment to promote or hinder the lifestyle behavior of interest. The external environment includes the remaining 3 outermost rings in the framework. The first of these 3, labeled *behavioral settings,* are the contexts, both physical and social, in which lifestyle behaviors occur. *Proximal leverage points* are "controllers of the structure and feature of the microenvironment that affect the choices related to the behavior of interest" (11). Finally, *distal leverage points *reflect macro-level influences on behavior and address systemwide factors such as the health care system, the information industry, the food industry, and the government. Application of this model to the qualitative transcripts allowed us to categorize participants' perceptions as either internal or external environmental influences on fruit and vegetable intake. In this report, the terms *intrinsic* and *extrinsic* are used to describe the individual (personal) and external (physical/social) environments, respectively.

## Results

The survey sample (N = 2,479) had a mean (SD) age of 52.8 (15.3) years, was predominantly female (72%), non-Hispanic white (75%), married (63%), and had a high-school diploma (87%). More than 44% of the sample had a total annual household income of less than $30,000. The mean  (SD) BMI was 29.4 (7.1), and the mean (SD) number of comorbid conditions was 3 (2) (Table 2). The most prevalent chronic conditions reported, aside from musculoskeletal diseases, were high blood pressure (45.3%), depression (30.8%), diabetes (18.7%), ulcer or stomach disease (gastroesophageal reflux disease or acid reflux and gastritis) (15.1%), and heart disease (14.8%). Some participants reported more than 1 of these conditions. Compared with the survey sample, the formative assessment sample of 32 participants was older (mean [SD] age, 56 [13.9] years), heavier (mean [SD] BMI, 32.2 [7.8]), poorer (66.7% had annual household incomes <$30,000), and less educated (68.8% had a high school diploma) (Table 2).

### Telephone survey results

Participants responded to 3 survey questions assessing food quality, variety, and affordability (Table 3). BMI and counts of comorbid conditions were associated with significant differences in perceptions about quality of fruits and vegetables available at the grocery store where participants regularly shopped. The number of comorbid conditions was also significantly related to perceptions of variety. For affordability, we found significant differences by income, education, BMI, poverty rate by census block group, and comorbid conditions (Table 3). Age was significant for all 3 questions.

### Formative results

Transcripts revealed a rich dataset of perceptions related to factors perceived to affect intake of fruits and vegetables. Findings from the community mappings helped to contextualize participants' perceptions. Photographs shown and discussed by participants during the sessions included images of farms, restaurants, kitchen spaces, convenience stores, gardens, buffet foods, and condiments, and generally supported participant perceptions. We used the framework by Booth and colleagues to categorize perceptions on the basis of related environmental factors highlighted in the framework and by the influence (barrier or facilitator) of the perception on dietary behavior (Table 4). Most perceptions were categorized as either a barrier or a facilitator. However, some had dual influences.

The following intrinsic factors were identified as barriers: food preferences, fatigue of taste buds for certain foods, life stresses, lack of forethought in meal planning, current personal health status, aging, and perceived impact of food on current chronic disease status. Intrinsic factors perceived as facilitators were the presence of chronic disease, lifetime experience related to intake of fruits and vegetables, preferences for certain fruits and vegetables, and personal or spousal health status. At the extrinsic level, participants reported the following factors as facilitators: availability of home gardens, low cost of foods at farm stands, and childhood exposure to fruits and vegetables. Perceived barriers included contradictory media messages related to nutrition and health outcomes, worksite food options, food availability, and food cost at grocery stores. Finally, participants reported the following as factors perceived to have an interactive effect: concerns about food safety and perceptions about the interaction between chronic disease status and social and environmental influences on behavior and health. For example, participants highlighted the interactions between physical fatigue due to changing work schedules or shifts and stresses resulting from trying to manage fatigue, work schedule, and personal dietary intake at home. Some participants perceived chronic disease status as a facilitator, whereas to others it was a barrier.

## Discussion

Intake of fruits and vegetables is a major factor in the prevention of chronic diseases. The continued increase in chronic disease and obesity and the corresponding increase in poor chronic disease outcomes require different approaches. Using survey methods, we found that this sample was significantly overweight and was affected by chronic diseases. The 2007 health profile of North Carolina residents indicates suboptimal nutritional practices. Reportedly, only 23% of residents consume the recommended daily intake of 5 or more fruits and vegetables, and 26% did not participate in leisure-time physical activity (3).

A recent survey that assessed the dietary habits of 1,788 people who were part of the 2005 enrichment of the NC-FM-RN reported that less than one-third had a daily intake of 3 or more servings of fruits and vegetables (22). These participants were not part of the NC-FM-RN cohort when our study was conducted. However, the mean BMI (29.6), which was calculated from self-reported weight and height (22), was similar to that of our sample. State-level data regarding participants' contextual perceptions are lacking and were not assessed by the earlier study. Nevertheless, a recent study in the United Kingdom has reportedly demonstrated a relationship between individuals' socioeconomic context, perceptions about food intake, and BMI status (23). Our study's use of formative research methods enabled us to provide significant information about a specific North Carolina population that complements the findings of other surveys on perceptions about the variety, affordability, and quality of foods available to participants and the impact of context on intake of fruits and vegetable.

We did find that differences in education, poverty level, chronic disease status, and income affected perceptions of affordability, which may affect individuals' intake. Affordability was perceived as a barrier for poorer participants. However, no racial differences were found. These findings suggest that economics or SES rather than race or culture may be the key variable differentiating participants' perceptions of the affordability of fruits and vegetables. In fact, studies indicate that income differences have a greater effect on diet quality than on overall energy intake between groups (24). We also found significant differences in BMI levels related to quality. A greater percentage of people with high BMI perceived the quality of the fruits and vegetables at their grocery store to be excellent compared to people with low BMI. Perhaps those with higher BMI are less discriminating and perceived all foods, including fruits and vegetables, to generally be of great quality. For categories of comorbid conditions, however, differences across all 3 variables (quality, variety, and affordability) were found between those with no chronic condition and those with several. People with no comorbid condition were less likely to rate the variety and quality of their foods as excellent and were somewhat likely to rate the affordability as excellent. This finding may reflect the association between comorbidity and disability and their combined impact on income and access. People with more comorbid conditions are potentially more likely to be disabled (25), which affects their income and therefore could affect their perception of the affordability of fruits and vegetables. This might account for why a lower percentage of people with more chronic conditions perceived fruits and vegetables as very affordable. Similarly, it is possible that those with no comorbid condition reflect a health-conscious group that has higher income and health expectations and are therefore more critical in their evaluations of the quality and variety of foods available to them. The lower percentage of people in this category who perceived that the quality and variety of fruits and vegetables available to them is excellent is consistent with this conclusion.

### Formative assessment

One potential limitation of this study is the small sample used for the formative assessment. Researchers, however, indicate that, regardless of qualitative methodology used (focus group or key informant interviewing), with adequate representation, a sample of 30 individuals is enough to uncover the perceptions of the majority of a population (26,27). Other limitations of the study are that intake of fruits and vegetables, nutrition behavior, and weight and height data (for BMI) were not directly measured. Hence, correlations and causality cannot be verified for these variables.

Participants' perceptions of intrinsic and extrinsic influences on intake of fruits and vegetables are consistent with those of other studies (23,28-30) but specifically indicate that in this sample, behaviors and attitudes toward fruits and vegetables are influenced by both intrinsic and extrinsic environmental factors. Furthermore, they provide grounds for reconsidering the focus of approaches to dietary interventions to see how salient they are in addressing the perceived needs for (or barriers to) behavior change in this population. Many interventions are based on findings from surveys that tend to focus on individuals' behaviors with less emphasis on context. Our results suggest that a complex web of factors and perceptions underpins participants' nutrition behaviors. Overall, the results suggest that interventions should use multifactorial approaches that acknowledge the contexts of individuals and their environmental limitations and should provide options that people can use to achieve healthy lifestyle habits. Interestingly, most of the perceptions described by the participants of this study were more intrinsic than extrinsic. This finding suggests that individually focused interventions would be useful in helping them with chronic disease risk management. Family practice settings, from which all the participants of this study were recruited, may be an ideal place to start because they can serve as important sources of information for health promotion and disease prevention (22,31-33). A recent study confirms that individually tailored messages can significantly affect the nutrition behaviors of people who visit family practice settings (33). However, researchers also indicate that the "time has come for interventionists (whether public health or primary care) to look more closely at context — the risk-laden conditions within communities — rather than limit the focus to individual risk factors" (34). Thus, the emphasis should not be limited to individually focused efforts but expanded to include linking individuals and family practice settings with available community-based resources. This effort is feasible for North Carolina; already several community-based programs promote healthy nutrition habits. One notable program is the Eat Smart Move More North Carolina statewide initiative (www.eatsmartmovemorenc.com). This program targets all North Carolinians and has offerings designed for a multitude of settings and audiences. These offerings can be individually tailored, and when executed properly, allow individuals to access resources that promote risk reduction by increasing healthy nutrition and physical activity practices.

The findings of this study indicate that many unfavorable intrinsic and some extrinsic factors are perceived to affect the intake of fruits and vegetables of this sample of North Carolinians. The perceptions evidenced are of concern because they are associated with behaviors that increase chronic disease risk. Options that would facilitate increased fruit and vegetable intake are needed, and family practice settings and community-based programs may be useful places to begin.

## Acknowledgments

This study was funded by National Institute of Arthritis and Musculoskeletal and Skin Diseases grant no. 5P60-AR49465-01. Dr Boyington was supported by the National Institute of Arthritis and Musculoskeletal and Skin Diseases grant no. 5P60-AR49465-04S1, National Institutes of Health, the National Center on Minority Health and Health Disparities grant no. R24 MD000167, and the Department of Health and Human Services, Agency for Healthcare Research and Quality R24 HS013353. Ms Remmes Martin was supported by the Carolina Program on Health and Aging Research Predoctoral Fellowship (National Institute on Aging grant no. 5-T32-AG00272), the Arthritis Foundation Doctoral Dissertation Award, and the ACR REF Health Professional Graduate Student Research Preceptorship Award.

We thank the following participating family practices in the North Carolina Family Medicine Research Network for their assistance: Black River Health Services, Burgaw; Bladen Medical Associates, Elizabethtown; Blair Family Medicine, Wallace; Cabarrus Family Medicine, Concord; Cabarrus Family Medicine, Harrisburg; Cabarrus Family Medicine, Kannapolis; Cabarrus Family Medicine, Mt. Pleasant; Chatham Primary Care, Siler City; CMC-Biddle Point, Charlotte; CMC-North Park, Charlotte; Community Family Practice, Asheville; Cornerstone Medical Center, Burlington; Dayspring Family Medicine, Eden; Family Practice of Summerfield, Summerfield; Goldsboro Family Physicians, Goldsboro; Henderson Family Medicine Clinic, Henderson; Orange Family Medical Group, Hillsborough; Person Family Medical Center, Roxboro; Pittsboro Family Medicine, Pittsboro; Prospect Hill Community Health Center, Prospect Hill; Robbins Family Practice, Robbins; and Village Family Medicine, Chapel Hill.

We also gratefully acknowledge Andrea Meier, PhD, for her technical and research assistance in developing study materials, training study investigators, and troubleshooting data analysis software problems; Thelma Mielenz, PhD, for her assistance with Table 2; and Matthew Morrison for attending to the logistics of planning and executing the focus groups and key informant interviews. Buncombe County Library, Henderson County Council on Aging, North Carolina Cooperative Extension — Bladen County Center, Person County Public Library, Rockingham County Public Library — Eden Branch, and Wayne County Public Library are also gratefully acknowledged for providing facilities for the focus group discussions. We also thank Diane Beth at the Physical Activity and Nutrition Branch of the North Carolina Department of Health and Human Services for her assistance in reviewing current nutrition-based health programs in North Carolina. Finally, we thank the participants in the study.

## Figures and Tables

**Table 1:** Individual (Intrinsic) and Community (Extrinsic) Environmental Determinants of Physical Activity and Eating Behavior, a North Carolina Family Medicine Research Network Study, 2004^a^

**Psychobiologic Core**	**Cultural**	**Social**	**Enablers of Choice**	**Behavioral Settings**	**Proximal Leverage Points**	**Distal Leverage Points**
Self-identities Pleasures Genetics Hierarchy of needs Physiology (physical health)	Habits Ethnic identities Beliefs Values Life experience	Social roles Life stage Interpersonal relationships Educational attainment Socioeconomic status	Social trends Seasonality Convenience Accessibility Situation or context – physical and social Source of information Cost Time Safety Knowledge	Home Food stores Health clubs Workplace Community activity providers Restaurants Religious, community, and nongovernmental organizations Parks, recreation centers, senior centers Vehicle for transport Shopping malls Neighborhood Daycare Local school	FamilyFood stores Local government Developers Property owners Restaurants and food outlets Recreation facilities Nongovernmental organizations Nonprofit providersCommunity Shopping malls Health care providers School board district Employer	Political advocacy/lobbying Food industry Transportation system Architecture and building codes Exercise, physical activity, sports industry Recreation industry Health care industry Education system Entertainment industry Labor-saving device industryInformation industry National government

a Adapted from reference 11.

**Table 2:** Demographic Characteristics of Survey (N = 2,479) and Formative Assessment Participants (n = 32), a North Carolina Family Medicine Research Network Study, 2004

**Participant Characteristics**	**Telephone Survey Participants^a^**	**Formative Assessment Participants^a^**
Mean age, y (SD)	52.8 (15.3)	56 (13.9)
Mean body mass index, kg/m^2^ (SD)	29.4 (7.1)	32.2 (7.8)
Mean no. of comorbidities (SD)	3 (2.2)	3 (1.7)
Female, %	72.2	71.9
Non-Hispanic white, %	75.4	65.6
Received high school diploma, %	86.7	68.8
Annual household income <$30,000, %	44.2	66.7
Married, %	62.6	62.5

a Excludes participants with incomplete demographic data.

**Table 3:** Perceptions of Quality, Variety, and Affordability of Fruits and Vegetables by Selected Characteristics Among Survey (N = 2,479) and Formative Assessment Participants (n = 32), a North Carolina Family Medicine Research Network Study, 2004^a^

Category	% of Survey Respondents^b^								

	Quality			Variety			Affordability		

	Excellent	Good	Fair/Poor	Excellent	Good	Fair/Poor	Excellent	Good	Fair/Poor
**Income, $**	*P* = .92			*P* = .48			*P* < .001		
<30,000	33.0	57.4	9.5	33.1	55.9	11.0	17.6	73.1	9.4
≥30,000	33.8	56.6	9.6	34.6	53.4	12.0	26.0	71.3	2.7
**Education**	*P* = .61			*P* = .73			*P* <.001		
<High school diploma	32.6	59.2	8.2	32.3	56.3	11.4	20.1	65.0	14.9
≥High school diploma	33.5	56.7	9.7	34.4	54.0	11.6	22.3	73.1	4.6
**Age, y**	*P *= .002			*P* <.001			*P* = .003		
≥60	36.8	55.5	7.7	39.7	51.9	8.4	24.3	67.8	7.9
46-59	33.7	58	8.3	34.4	54.1	11.5	21.2	73.1	5.6
≤45	29.8	57.9	12.3	28.6	57	14.4	21	75.1	3.9
**Poverty rate by census block group**	*P* = .67			*P* = .44			*P* = .02		
>12.3%	32.5	57.8	9.7	32.8	55.8	11.4	20.5	71.9	7.6
≤12.3%	34.3	56.7	9.0	35.5	53.3	11.2	23.0	72.1	4.9
**Race/ethnicity**	*P* = .11			*P* = .16			*P* = .11		
African American	37.7	54.2	8.1	34.6	56.6	8.8	18.9	74.4	6.8
Non-Hispanic white	32.5	57.7	9.8	34.5	53.6	12.0	23.4	70.9	5.7
**Body mass index, kg/m^2^**	*P* = .03			*P* = .90			*P* = .58		
>30	37.0	53.3	9.7	35.5	53.2	11.3	20.9	72.8	6.4
25-30	32.5	58.4	9.1	34.7	54.4	10.9	22.2	72.8	5.0
<25	29.5	60.1	10.3	33.2	54.9	11.8	23.5	70.4	6.0
**Comorbidity^a^**	*P* = .001			*P* = .01			*P* < .001		
>3	32.7	55.8	11.5	33.8	53.6	12.6	17.9	71.3	10.8
1-3	35.7	56.4	7.9	36.3	52.9	10.7	24.8	71.2	4.0
0	25.8	62.8	11.4	26.3	61.1	12.6	20.8	75.4	3.8

a Comorbid counts were determined by summing counts of 18 separate conditions including cancer, heart disease, and diabetes.

b *P* values are for the χ^2^ test (α = .05).

**Table 4:** Perceptions of Formative Assessment Participants (n = 32) on Quality, Variety, and Affordability of Fruits and Vegetables by Environmental Factors and Influence (Barriers or Facilitators) on Dietary Behavior, a North Carolina Family Medicine Research Network Study, 2004

**Perception**	**Sample Quotation^a^**	**Applicable Environmental Factors^b^**	**Enablers of Choice^c^**	**Effect^d^**
Factors associated with aging hinder intake of fruits and vegetables.	"I guess to be very honest with you, teeth — that's the hardest part, things I used to eat when I was younger like apples and things like that, I can't eat. It's a little more difficult with false teeth." (KI1552)	Physiology (physical health), genetics, life stage	Situation or context	Barrier
Current chronic disease status can hinder intake of fruits.	"Well, when my tomatoes is coming in I was eating tomatoes. As far as fruits go, most of them I eat come in the can, but I've sort of backed away from sugar and they put sugar and syrup and all that in the fruits now. So I had to back off of them." (KI2305)	Physiology (physical health), genetics	Knowledge, safety, source of information, food industry	Barrier
Current chronic disease status can promote intake of fruits and vegetables.	"Well, because I'm diabetic, I keep a food diary of everything I eat, which helps a little bit with keeping the calorie count of what I eat .... We try to, we eat fruits and vegetables, we eat quite a few vegetables and try to stay away from the red meat." (KI I552)	Physiology	Knowledge, health care providers, health care industry	Facilitator
Early childhood exposure to vegetables promotes continued preference for and consumption of fruits and vegetables.	"To me, a well-balanced diet was something I grew up with, and we still have that even though it's just the 2 of us. We have a salad almost every day, we have a hot vegetable, [and] we have our starch and our protein." (FG-H)	Pleasures, self-identities, habits, values, life experience	Situation or context	Facilitator
Fatigue of taste buds and conditioning of palate limits consumption of fruits and vegetables.	"To keep from eating the same vegetable all the time, sometime I choose not to eat any at all. So that's what keeps me from eating vegetables all that much, I don't like a wide variety of them like I do the fruit." (KI3201)	Pleasures	Habits	Barrier
Work-related stress promotes unhealthy eating patterns.	"It definitely impacts my eating habits. Through stress and stress eating and through, I guess, sort of how much I work in terms of getting off late and being tired and not having time to fix a fresh, healthy dinner." (KI2882)	Hierarchy of needs, social roles, life stage, interpersonal relationships, socioeconomic status	Knowledge, situation or context	Barrier
Lack of forethought and inadequate planning and preparation decreases consumption of fruits and vegetables in daily diet.	"Definitely. The fruits are a real struggle for me because I'm not one to prepare fruit. Like I hate peeling oranges and things like that." (KI2882)	Hierarchy of needs, habits	Knowledge, time	Barrier
Laziness affects daily intake of fruits and vegetables.	"And, to me, I'll just eat more of one thing than try to fix a variety of vegetables, because I'm worth it, but don't think it's worth the time." (FG-D)	Values, beliefs	Knowledge, convenience	Barrier
Easy access to fast-food restaurants affects nutrition behavior.	"Why would they want to cook when you have every fast food restaurant possible within a quarter mile of each other? They have no incentive to cook." (FG-AP)	Restaurants and food outlets, life stage, values	Accessibility, convenience	Barrier
Fast food is a necessity when people are busy.	"A lot of people do fast food because it's fast and because people are busy, and because they're working and because you're going here [and] there." (FG-B)	Restaurants and food outlets, life stage, beliefs, values	Time, cost, social trends, accessibility, convenience	Barrier
Impact of food on disease symptoms influences intake.	"Yeah. Cause I think steak makes my hand hurt worse. I think it do. And pizza." (FG-BP)	Physiology	Knowledge, safety, source of information	Barrier
Spouses are a positive influence on intake of fruits and vegetables.	"Yeah, we try to eat as healthy as we can. My wife especially likes to eat healthy. She cooks right many vegetables." (KI4141)	Self-identities, interpersonal relationships	Situation or context	Facilitator
Personal value of cooking at home compared to eating out affects intake.	"I cook because I buy groceries .... I don't eat out. A lot of times people say, come on, let's eat out, but I say no, I've already bought my groceries, so I cook ... from scratch." (FG-AP)	Values, habits, interpersonal relationships, social roles	Time, cost, knowledge	Facilitator
Benefits and costs of maintaining a garden compared to purchasing at food stands affects intake of fruits and vegetables.	"I had 2 gardens, but I gave it up because it wasn't worth the effort for just me and my husband. These farms selling vegetables, I go down and pick 'em." (FG-AP)	Values, social roles, life stage	Accessibility, cost, time, convenience	Facilitator and barrier
Perceived food safety risks such as antibiotics and pesticides in foods influence consumption decisions.	"And if you get meat that don't have hormones in them or antibiotics or anything like that, they are much higher." (FG-D)	Hierarchy of needs, physiology, values, beliefs	Knowledge, source of information, safety, social trends, cost	Barrier
Inconsistency in media messages and contradictions in health professionals' opinions affect choices and intake.	"Year ago they tried to tell you that the margarine was better for you, now they're back ... they can't decide .... Yeah, one year eggs is terrible for you. The next year they're telling you to eat as many as you can." (FG-AP)	Health care industry, information industry, health care providers, food industry	Knowledge, source of information, safety, social trends	Barrier
Inflexible work schedules promote poor dietary habits.	"When you teach at school, you don't have a choice of going out to lunch. So that was not good for my health because when the kids left at 3 o'clock, I was starving and would eat whatever." (FG-D)	Workplace, local school	Time, accessibility, convenience	Barrier
For employed workers, food options at worksites influence intake.	"We have a kitchen where you can prepare your food and where they provide snack[s] for you .... Yeah, like cakes ... but it would be like cakes, cookies, pies, homemade stuff like." (KI2780)	Community, workplace	Accessibility, convenience	Barrier
Lack of finances affects purchasing of fruits and vegetables.	"Well, in my case, it would be finances." (KI3559)	Socioeconomic status	Cost	Barrier
Lack of transportation decreases access to fruit and vegetables.	"And of course if you don't have a car, it's very difficult or if you're not driving to get out to the fruit stands and the vegetable stands." (FG-H)	Socioeconomic status	Cost, time, accessibility, situation or context	Barrier
Prices at farmers' market are lower and therefore influence purchase of fruits and vegetables.	"You can get good prices at the fruit stand." (FG-AP)	Food stores, farmers' market	Accessibility, seasonality, cost, socioeconomic status	Facilitator
Fatigue and long work hours hinder healthy nutrition behavior.	"Because you can't eat but at certain times. Most of the time by the time you eat, you have to go to bed. And it kind of messes you up trying to eat and go to sleep. Makes you gain weight." (KI3173)	Hierarchy of needs, physiology, home	Social roles, life stage, socioeconomic status, time	Barrier
Variability in (shift) work schedules alters eating patterns.	"Oh yeah, well, there were occasions when you normally take a lunch break, but you couldn't ... You had to wait until a later time ... your schedule. It would just steadily change." (KI3559)	Life stage, social role, workplace, community	Time, situation or context	Barrier
Economic constraints resulting from chronic disease status negatively influence purchase and intake of fruits and vegetables.	"Another problem that we have since we're both disabled, we live on a limited income so you can't always get what you need." (FG-H)	Socioeconomic status, local government, (national) government	Cost, accessibility	Barrier
Low personal income status affects intake of fresh fruits and vegetables.	"The only thing that makes it hard is money wise." (KI3720)	Socioeconomic status	Cost, knowledge	Barrier
Healthy foods are expensive.	"The reason I laugh is because the healthy food is expensive. It's all junk food that is reasonably priced. But most of the healthy food is very expensive." (KI3720)	Food stores, food industry	Cost, knowledge	Barrier

a Excerpted quotes from focus group (FG) and key informant (KI) or individual interview transcripts.

b Environmental factors from the framework by Booth et al (11), which are evidenced by the quotation.

c Applicable enablers identified from the framework.

d Impact of the reported perception on intake.
